# A method to improve genetic analysis of lung cancer samples

**DOI:** 10.1111/resp.14114

**Published:** 2021-07-11

**Authors:** Tadashi Sakaguchi, Akemi Iketani, Kazuki Furuhashi, Yuki Nakamura, Yuta Suzuki, Kentaro Ito, Kentaro Fujiwara, Yoichi Nishii, Koji Katsuta, Osamu Taguchi, Osamu Hataji

**Affiliations:** ^1^ Respiratory Center Matsusaka Municipal Hospital Matsusaka Japan; ^2^ Matsusaka Municipal Hospital Pathology Department Matsusaka Japan

**Keywords:** genetic analysis, lobectomy, next‐generation sequencing, non‐small cell lung cancer

*To the Editors*:

The Oncomine Dx Target Test (ODxTT) (Ion Torrent PGM Dx Sequencer; Thermo Fisher Scientific) is a next‐generation sequencing panel, which detects 46 genes on DNA and RNA isolated from formalin‐fixed and paraffin‐embedded (FFPE) specimens using the Ion PGM™ Dx System. This test enables comprehensive biomarker testing for non‐small cell lung cancer (NSCLC),^1^ and was approved as a companion diagnostic for targeted therapies by the US Food and Drug Administration in June 2017, and the Ministry of Health, Labor and Welfare of Japan in February 2019.^2^ Although the quantity of tumour cells in surgical samples is greater than in small biopsy samples, the quality of nucleic acids, especially RNA, is prone to deterioration in surgical samples. Therefore, we experienced invalid RNA analysis results due to a failure to meet the RNA sample QC (quality control) metrics, especially in lobectomy samples. We believed the causes were the delay until the beginning of formalin fixation and insufficient formalin fixation. We retrospectively assessed the influence of a modified lobectomy sample preparation process on nucleic acid quality and the success rate of the ODxTT.

This retrospective study was conducted at Matsusaka Municipal Hospital, Japan. We reviewed the electronic data from consecutive patients diagnosed with NSCLC whose FFPE surgical lung samples had been submitted for ODxTT between August 2019 and August 2020. Samples collected in other hospitals and archived samples were excluded. Clinical data assessments included surgical method, pathological findings, genetic test results and quantity and quality of nucleic acids.

Figure 1 shows a flow chart of the FFPE sample preparation process for lung surgery. The limited resection samples, including lung segmentectomy and wedge resections, were immediately put in 10% neutral buffered formalin (NBF) after sampling for intraoperative rapid diagnosis (IRD) for 24–48 h at room temperature (RT). The lobectomy samples taken between August 2019 and December 2019 were stored in a refrigerator at 4°C for less than 3 h after sampling for IRD and then put in 10% NBF for 24–48 h at RT (early period process), resulting in a longer time to formalin fixation. From January 2020, we changed the process for lobectomies, taking 10 mm × 10 mm samples in tumour‐rich areas concurrently when sampling for IRD for the ODxTT. These samples were put in 10% NBF immediately after sampling (later period process). Formalin‐fixed tissues were embedded in paraffin to create FFPE blocks. For the ODxTT, 5–10 slide‐mounted 5‐μm sections of the surgical samples were submitted to LSI Medience Laboratories (Tokyo, Japan). LSI Medience Laboratories performed the ODxTTs based on Thermo Fisher's Ion AmpliSeq technology.^2^ To assess the quality of nucleic acids, we evaluated DNA integrity number for DNA quality and total mappable reads (TMR), which was used for the evaluation of RNA sequencing quality on ODxTT, and DV200 for RNA quality.

**FIGURE 1:**
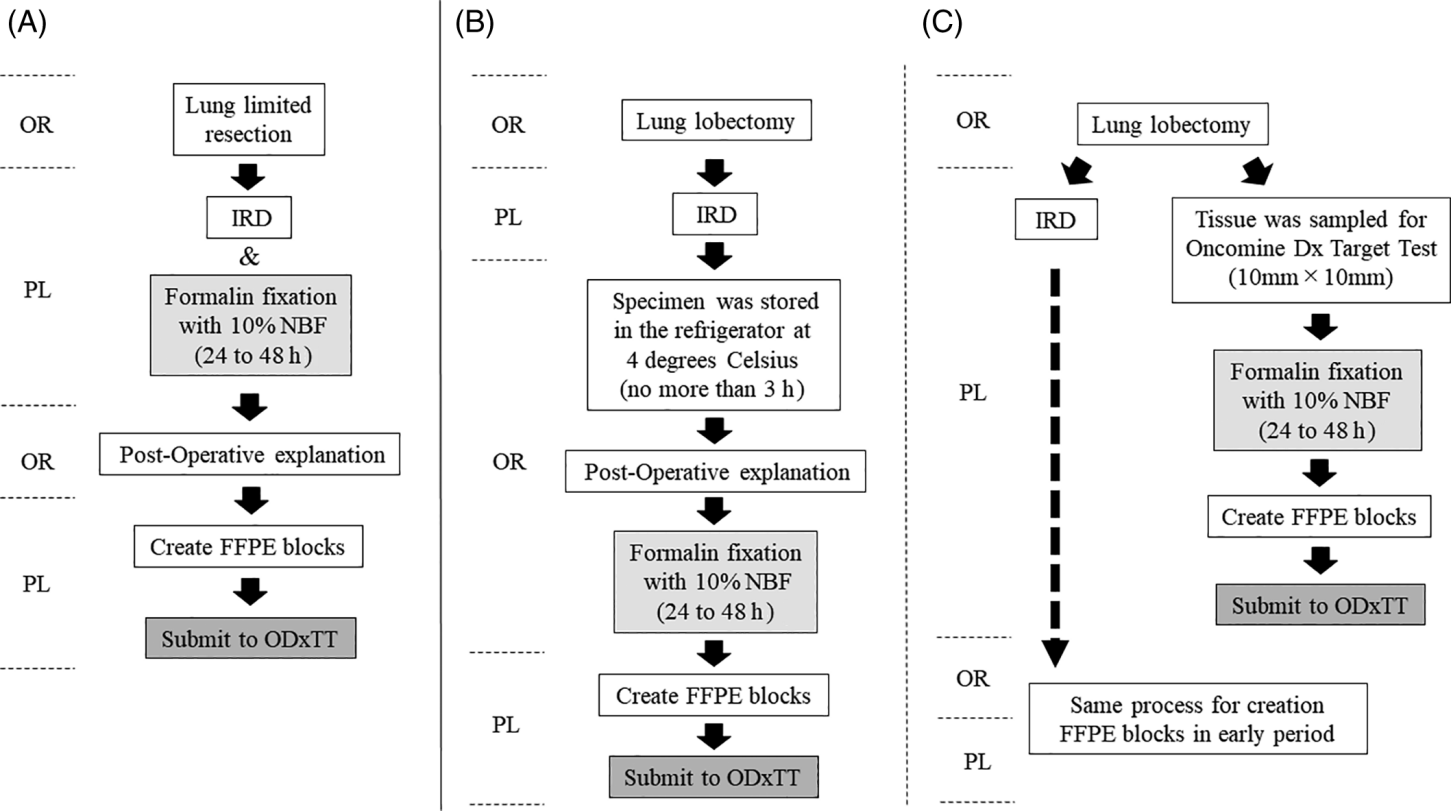
Flow chart of FFPE sample preparation process on lung surgery. (A) Process on limited resection. (B1) Early period process on lobectomy. (B2) Later period process on lobectomy (modified FFPE sample preparation process). FFPE, formalin‐fixed and paraffin‐embedded; IRD, intraoperative rapid diagnosis; NBF, neutral buffered formalin; ODxTT, Oncomine Dx Target Test; OR, operating room; PL, pathology laboratory

We compared the success rates of the ODxTT between the early and later periods based on the surgical method and quantity and quality of nucleic acids for lobectomy samples. Results of the ODxTT were considered successful if all results were valid for the four companion diagnostic genetic targets approved in Japan: *EGFR* (epidermal growth factor receptor), *ALK* (anaplastic lymphoma kinase), *ROS1* (ROS proto‐oncogene 1, receptor tyrosine kinase) and *BRAF* V600E (B‐Raf proto‐oncogene, serine/threonine kinase). Results were considered unsuccessful if the sample did not pass the nucleic acid concentration threshold, or if one or more of the aforementioned results were invalid.

Statistical analyses were performed using Student's *t*‐test and Fisher's exact test for continuous and categorical variables. Statistical analyses were performed using SPSS software, version 26.0 (SPSS Inc., Chicago, USA). *p*‐Values of less than 0.05 were considered statistically significant.

A total of 71 samples were identified for analysis. Eighteen patients received lung surgery in the early period, including 11 lobectomies and seven limited resections. Fifty‐three patients were included in the later period: 25 lobectomies and 28 limited resections.

The success rate of the ODxTT, and the quantity and quality of nucleic acids by operative procedure and period are shown in Table 1. The success rate of lobectomy samples in the later period (96%) was significantly better than in the early period (55%) (*p* = 0.006). The quantity of DNA and RNA, and the quality of DNA were comparable between the two periods. For the quality of RNA, TMR above the threshold (≥5000) was higher in the later period (96% vs. 64%, *p* = 0.023). DV200 was also numerically higher in the later period, although not statistically significant.

**TABLE 1:** The success rate of the ODxTT, and the quantity and quality of nucleic acids by surgical method and period

	Lobectomy, *n* = 36					Limited resection *n* = 35				
	Early period, *n* = 11	%	Later period, *n* = 25	%	*p*‐Value	Early period, *n* = 7	%	Later period, *n* = 28	%	*p*‐Value
Results of ODxTT										
Success of analysis	6	55%	24	96%	0.006*	7	100%	25	89%	>0.999
Not passing the nucleic acid concentration threshold	0	0%	0	0%		0	0%	0	0%	
Invalid results for all DNA (*EGFR*, *BRAF*)	0	0%	0	0%		0	0%	0	0%	
Invalid results for all RNA (*ALK*, *ROS1*)	4	36%	1	4%		0	0%	3	11%	
Invalid results for subset of DNA and RNA	1	9%	0	0%		0	0%	0	0%	
Quantity and quality on DNA										
Mean quantity of DNA (μg) (95% CI)	25.8	20.1–31.5	24.6	18.1–31.1	0.824					
Mean DIN (95% CI)	5.9	5.8–6.0	5.8	5.6–6.0	0.133					
Quantity and quality on RNA										
Mean quantity of RNA (μg) (95% CI)	18.1	15.1–21.0	21.2	17.7–24.7	0.247					
Mean TMR (×10^3^) (95% CI)	47.9	20.3–75.5	62.9	35.2–90.0	0.489					
TMR above the threshold (≥5000)	7	64%	24	96%	0.023*					
Mean DV200 (95% CI)	39.4	23.1–55.8	49.1	37.9–60.4	0.317					
Category of DV200										
<30% (too degraded)	2	18%	4	16%						
30%–50% (low)	7	64%	11	44%						
50%–70% (medium)	0	0%	2	8%						
>70% (high)	2	18%	8	32%						

Abbreviations: ALK, anaplastic lymphoma kinase; BRAF, B‐Raf proto‐oncogene, serine/threonine kinase; DIN, DNA integrity number; EGFR, epidermal growth factor receptor; ODxTT, Oncomine Dx Target Test; ROS1, ROS proto‐oncogene 1, receptor tyrosine kinase; TMR, total mappable reads.

^*^ *p*‐Values of <0.05 were considered statistically significant.

The lower success rate of the ODxTT for lobectomy samples in the early period was due to invalid RNA analysis results. It suggested that RNA analysis required more sufficient fixation than DNA to deactivate RNase completely.^3^ Focusing on the differences between the FFPE preparation processes for lobectomies and limited resections, we hypothesized that the lower success rate in the early period was due to either deterioration of RNA resulting from the delay until the beginning of formalin fixation, or insufficient formalin fixation caused by large specimen size and a delay of formalin infiltration due to the sample being refrigerated. The infiltration rate of formalin was about 1 mm/h; therefore, large specimens were prone to insufficient formalin fixation. Refrigeration of the excised organ is useful for preservation of DNA and RNA; however, formalin fixation at low temperatures slows the formalin infiltration rate. Therefore, we modified the FFPE preparation process of lobectomy samples as mentioned above to address both possible causes of RNA deterioration. After modification, the success rate significantly improved compared with the early period process, indicated by improved RNA quality. Although Japanese guidelines for the handling of pathological tissue samples for genomic research allow surgically resected material to be kept refrigerated (4°C) until formalin fixation for up to 3 h to prevent the deterioration of RNA quality,^3^ our data suggested that immediate formalin fixation should be emphasized to refine RNA quality. This study was a relatively small retrospective study; therefore, further evaluations with larger cohorts are required. In conclusion, our modified lobectomy sample preparation process could improve the success rate of the ODxTT due to refined RNA quality.

## AUTHOR CONTRIBUTIONS

**Akemi Iketani:** Conceptualization; data curation; investigation; methodology; project administration; resources; validation; visualization. **Kazuki Furuhashi:** Data curation; project administration; resources; software. **Yuki Nakamura:** Data curation; investigation; resources; visualization. **Yuta Suzuki:** Data curation; investigation; project administration; resources; software. **Kentaro Ito:** Conceptualization; formal analysis; methodology; supervision; validation; writing‐review & editing. **Kentaro Fujiwara:** Investigation; methodology; resources; supervision; validation; visualization; writing‐review & editing. **Yoichi Nishii:** Conceptualization; data curation; investigation; resources; validation; visualization; writing‐review & editing. **Koji Katsuta:** Conceptualization; data curation; investigation; methodology; resources; supervision; writing‐review & editing. **Osamu Taguchi:** Conceptualization; funding acquisition; supervision; validation; writing‐review & editing. **Osamu Hataji:** Conceptualization; funding acquisition; project administration; supervision; validation; writing‐review & editing. **Tadashi Sakaguchi:** Conceptualization; data curation; formal analysis; investigation; methodology; project administration; resources; validation; writing‐original draft.

## CONFLICT OF INTEREST

Matsusaka Municipal Hospital received research grant funding from Novartis, GlaxoSmithKline, AstraZeneca, Daiichi Sankyo, Bayer and Boehringer Ingelheim. Kentaro Ito has received speaker fees as honoraria from Eli Lilly Japan, Chugai, AstraZeneca, MSD, Boehringer Ingelheim Japan, Ono and Pfizer Japan. Osamu Taguchi received speaker fees as honoraria from AstraZeneca. Osamu Hataji received speaker fees as honoraria from Novartis Pharma, AstraZeneca and Boehringer Ingelheim Japan. The remaining authors declare that they have no conflict of interest.

## HUMAN ETHICS APPROVAL DECLARATION

This study was approved by the institutional review board of Matsusaka Municipal Hospital (Approval date: 20 April 2020; IRB number J‐76‐200410‐5‐2) and was carried out in accordance with the Declaration of Helsinki. We obtained informed consent with and opt‐out choice in accordance to the Ethical Guidelines for Medical and Health Research Involving Human Subjects from Japanese government.
